# Modeling the Residential Infiltration of Outdoor PM_2.5_ in the Multi-Ethnic Study of Atherosclerosis and Air Pollution (MESA Air)

**DOI:** 10.1289/ehp.1104447

**Published:** 2012-02-22

**Authors:** Ryan W. Allen, Sara D. Adar, Ed Avol, Martin Cohen, Cynthia L. Curl, Timothy Larson, L.-J. Sally Liu, Lianne Sheppard, Joel D. Kaufman

**Affiliations:** 1 Faculty of Health Sciences, Simon Fraser University, Burnaby, British Columbia, Canada; 2 Department of Epidemiology, University of Michigan, Ann Arbor, Michigan, USA; 3 Department of Preventive Medicine, University of Southern California, Los Angeles, California, USA; 4 Department of Environmental and Occupational Health Sciences, and; 5 Department of Civil and Environmental Engineering, University of Washington, Seattle, Washington, USA; 6 Institute of Social and Preventive Medicine, University of Basel, Basel, Switzerland; 7 Department of Biostatistics,; 8 Department of Epidemiology, and; 9 Department of Medicine, University of Washington, Seattle, Washington, USA

**Keywords:** air exchange, attenuation, deposition, exposure misclassification, penetration, ventilation

## Abstract

Background: Epidemiologic studies of fine particulate matter [aerodynamic diameter ≤ 2.5 μm (PM_2.5_)] typically use outdoor concentrations as exposure surrogates. Failure to account for variation in residential infiltration efficiencies (*F*_inf_) will affect epidemiologic study results.

Objective: We aimed to develop models to predict *F*_inf_ for > 6,000 homes in the Multi-Ethnic Study of Atherosclerosis and Air Pollution (MESA Air), a prospective cohort study of PM_2.5_ exposure, subclinical cardiovascular disease, and clinical outcomes.

Methods: We collected 526 two-week, paired indoor–outdoor PM_2.5_ filter samples from a subset of study homes. PM_2.5_ elemental composition was measured by X-ray fluorescence, and *F*_inf_ was estimated as the indoor/outdoor sulfur ratio. We regressed *F*_inf_ on meteorologic variables and questionnaire-based predictors in season-specific models. Models were evaluated using the *R*^2^ and root mean square error (RMSE) from a 10-fold cross-validation.

Results: The mean ± SD *F*_inf_ across all communities and seasons was 0.62 ± 0.21, and community-specific means ranged from 0.47 ± 0.15 in Winston-Salem, North Carolina, to 0.82 ± 0.14 in New York, New York. *F*_inf_ was generally greater during the warm (> 18°C) season. Central air conditioning (AC) use, frequency of AC use, and window opening frequency were the most important predictors during the warm season; outdoor temperature and forced-air heat were the best cold-season predictors. The models predicted 60% of the variance in 2-week *F*_inf_, with an RMSE of 0.13.

Conclusions: We developed intuitive models that can predict *F*_inf_ using easily obtained variables. Using these models, MESA Air will be the first large epidemiologic study to incorporate variation in residential *F*_inf_ into an exposure assessment.

Epidemiologic studies have consistently linked exposure to particulate matter (PM) air pollution with adverse health effects (Pope and Dockery 2006). Early studies of long-term exposure measured concentrations at relatively few locations to investigate the health impacts of concentration differences between cities. Recent studies have used more sophisticated methods to improve the spatial resolution of exposure estimates (Health Effects Institute 2010).

Despite these improvements, most epidemiologic studies still assume that outdoor concentrations represent personal exposure to PM of outdoor origin, even though individuals spend most of their time indoors. Several studies have demonstrated that fine PM [aerodynamic diameter ≤ 2.5 μm (PM_2.5_)] infiltration efficiency (*F*_inf_), defined as the fraction of the outdoor concentration that penetrates indoors and remains suspended, varies between communities, between homes, and over time within homes (Chen and Zhao 2011). Failure to account for this potential source of exposure variation in epidemiologic studies may be a source of exposure misclassification that could limit our ability to accurately estimate the health risks of long-term PM exposure (Sarnat et al. 2007).

Multiple methods have been developed for estimating *F*_inf_, a variable that depends on the air exchange rate, PM loss rate (the rate at which PM is removed from the air by deposition, filtration, and so forth), and penetration efficiency (the fraction of PM that penetrates the building envelope as outdoor air comes indoors). The use of sulfur or sulfate as an outdoor PM_2.5_ tracer is the most common method for estimating *F*_inf_. Sulfur is a useful tracer because it has few indoor sources and has infiltration characteristics roughly similar to PM_2.5_ (Sarnat et al. 2002). Therefore, in the absence of indoor sulfur sources, the indoor/outdoor (I/O) sulfur ratio provides a good estimate of *F*_inf_ for nonvolatile PM_2.5_ components.

Unfortunately, methods for estimating *F*_inf_ in residences require indoor and outdoor pollution sampling, which makes estimating *F*_inf_ among large populations infeasible. To overcome this challenge, some investigators have developed *F*_inf_ prediction models (Clark et al. 2010; Hystad et al. 2009; Koenig et al. 2005; Meng et al. 2009). Although the models have shown promise, they have generally been developed for individual cities using relatively small sample sizes and therefore may not be transferable to other locations.

The Multi-Ethnic Study of Atherosclerosis and Air Pollution (MESA Air) is a prospective cohort study of the relationship between long-term exposure to PM_2.5_, subclinical cardiovascular disease, and clinical outcomes (Kaufman et al. 2012). More than 6,000 participants between 45 and 84 years of age will be followed over approximately 10 years for cardiovascular disease events and mortality, and subcohorts are being assessed for subclinical cardiovascular disease progression. In this article, we describe the development of models for predicting *F*_inf_ on a 2-week basis for every study home. These *F*_inf_ estimates will be combined with outdoor PM_2.5_ concentration estimates and individual time–location patterns to estimate every participant’s long-term exposure to PM_2.5_ of outdoor origin (Cohen et al. 2009).

## Methods

*Study design.* Most MESA Air participants were recruited from the main MESA study (Bild et al. 2002), which includes six communities: Baltimore City and Baltimore County, Maryland; Chicago, Illinois; Forsyth County (Winston-Salem), North Carolina; Los Angeles County, California; New York, New York; and St. Paul, Minnesota. To enhance exposure heterogeneity in MESA Air, additional participants were recruited from two areas in the Los Angeles basin (coastal Los Angeles and an area ~ 90 km inland near Rubidoux in western Riverside County) and in Rockland County, New York (~ 40 km north of New York City). For this analysis, the additional Los Angeles area participants were combined with Los Angeles County participants recruited from MESA. Primarily because of differences in housing characteristics, Rockland County participants were considered separately from New York City participants. Thus, we considered seven study communities. All of the participating centers’ institutional review boards approved the study, and all study participants gave written informed consent before data collection.

The exposure assessment approach in MESA Air has been previously described (Cohen et al. 2009). The overarching goal is to develop accurate estimates of participants’ outdoor-origin PM_2.5_ exposure over the 10-year study period (Kaufman et al. 2012). It is not feasible to make ongoing residential or personal concentration measurements for the entire study. Therefore, MESA Air makes use of a modeling approach, in which a limited number of measurements in each study community and at a subset of participants’ homes are used to develop models to estimate both outdoor concentrations and *F*_inf_ across the entire study population. Other reports have focused on the outdoor concentration prediction approaches (Sampson et al. 2011; Szpiro et al. 2010); this article addresses the estimation of *F*_inf_ for this cohort.

*Data collection.* Every participant completed the MESA Air Questionnaire (henceforth “main questionnaire”) at recruitment, and this questionnaire was repeated during follow-up calls when participants indicated a change of residence. This questionnaire was used to gather information on residence characteristics and resident behaviors related to *F*_inf_, including building type, presence/use of air conditioning (AC), window opening, and use of air filters/cleaners. For behaviors that vary seasonally, we asked participants about typical behavior in the previous summer and winter.

Between March 2006 and July 2008, a subset of homes underwent I/O residential pollution sampling. Details of sample collection can be found in Cohen et al. (2009). In brief, outdoor sampling equipment was usually placed in the participant’s back yard or patio, away from all structures. When this was not possible (e.g., in the case of high-rise apartments), outdoor samplers were extended approximately 1 m out an available window and the window sealed with weather stripping. Indoor sampling equipment was placed in the main activity room away from pollution sources and ventilation systems. Homes were selected to cover the geographic area of each community and to represent a range of proximities to major roads, and only nonsmoking households were selected for I/O sampling, because smoking is a weak indoor sulfur source (Koutrakis et al. 1992). Each sampling period was nominally 2 weeks in duration, and many homes were monitored twice, usually in different seasons. The concentrations of 48 elements in the PM_2.5_ Teflon filter samples were quantified by X-ray fluorescence (Cooper Environmental Services, Portland, OR, USA). *F*_inf_ was calculated as the I/O ratio of 2-week average sulfur concentrations. The estimated precision of sulfur measurements (calculated as the relative percent difference of duplicate samplers divided by _√_^–^2) was 3.7%.

Participants whose homes had paired I/O sampling were also asked to complete an infiltration questionnaire, for which the completion rate was approximately 90%. Unlike the main questionnaire, the infiltration questionnaire focused on residence characteristics and resident behaviors during the 2-week period of I/O sampling. In addition, the infiltration questionnaire asked questions about potential indoor sources of PM_2.5_ (e.g., cooking) and sulfur (e.g., kerosene heaters).

All MESA Air home addresses were geocoded based on the Dynamap 2000 TeleAtlas road network (TeleAtlas, Menlo Park, CA, USA) using ArcGIS (version 9.2; ESRI, Redlands, CA, USA) (Cohen et al. 2009), and distances to the nearest major roads were calculated. Outdoor temperatures and precipitation during each 2-week period were obtained from the National Oceanic and Atmospheric Administration (2011).

*Data analysis and model building.* After data cleaning [described in detail in Supplemental Material, p. 3 (http://dx.doi.org/10.1289/ehp.1104447)], there were 526 I/O sulfur pairs (from 353 homes) for analysis and model building. For each valid *F*_inf_ observation, we estimated the contribution of infiltrated and indoor-generated PM_2.5_ to the total indoor concentration (described in detail in Supplemental Material, p. 3). Because our goal was to predict *F*_inf_ across the MESA Air cohort, we constructed our models using predictors that were available for every participant (henceforth “generalizable models”). We constructed season-specific models under the assumption that the *F*_inf_ predictors and their model coefficients would vary between seasons. To explore the consistency of predictors across communities, we first constructed season-specific *F*_inf_ models for each community before developing season-specific models using data from all communities. We categorized each 2-week period into a “warm” or “cold” season based on the average outdoor temperature (> 18°C and ≤ 18°C, respectively). We used 18°C as the cutoff because it was supported by the data (see Supplemental Material, Figure 1) and because it is commonly used in heating- and cooling-degree day calculations (Quayle and Diaz 1980).

**Figure 1:**
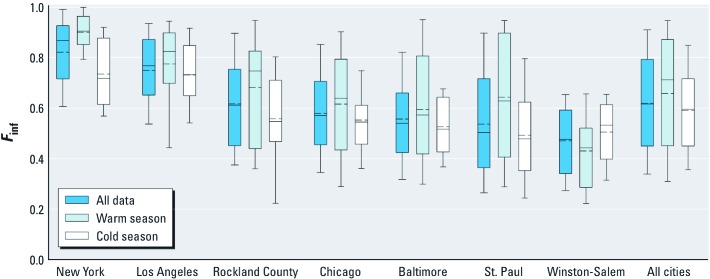
Distributions of 2-week average *F*_inf_ by community and season. Communities are shown in order of decreasing median *F*_inf_. Solid lines in boxes represent median values; dashed lines in boxes represent mean values; boxes represent 25th and 75th percentiles; whiskers represent 10th and 90th percentiles; outliers are not shown. The number of observations and homes for each community/season is given in Table 1.

We focused on predictors that have been previously associated with *F*_inf_, including outdoor temperature, building type, air cleaner/filter use, AC use, window opening, and use of forced-air heat. A correlation between residence age and *F*_inf_ has also been reported (Lachenmyer and Hidy 2000); however, building age was not known or not reported for nearly 13% of the MESA Air homes, so we did not include it as a potential predictor. We also evaluated the presence of an attached garage, double-pane windows, and storm windows as potential predictors of *F*_inf_, although these variables have not been associated with *F*_inf_ previously. In addition, proximity to major roads was included as a potential predictor because roadway noise might be correlated with window opening (Ohrstrom et al. 2006) and/or window quality (Klaeboe et al. 2004). Some potential predictors (e.g., window opening frequency) were coded as both an ordinal variable and as several binary variables with different cut-points. Outdoor temperature was coded as both continuous and binary with different cut-points. In total, we screened 84 potential predictors of *F*_inf_ in the generalizable models. We also considered several interactions with outdoor temperature. The details of the model building procedure are presented in the Supplemental Material, p. 4 (http://dx.doi.org/10.1289/ehp.1104447).

Generalizable model performances were assessed, and the “best” models were selected, using a 10-fold cross-validation (CV) method (Hastie et al. 2001) Each data set (season) being modeled was divided into 10 approximately equal-size groups. Because some homes were monitored twice in the same season, both measurements from a single home in a given season were placed in the same group. The model was then fit based on data from nine groups, and the estimated coefficients were used to predict *F*_inf_ for all observations in the excluded group. This procedure was repeated until predictions for all groups had been generated using SAS software (version 9.3; SAS Institute Inc., Cary, NC, USA). We calculated the CV *R*^2^ and the CV root mean square error (RMSE) by comparing predicted and measured *F*_inf_. To assess the potential for the models to predict *F*_inf_ outside of MESA Air, we also conducted a more conservative leave-one-community-out CV. Unless otherwise stated, CV results will be those from the 10-fold CV.

We evaluated the models’ robustness and representativeness in three ways. First, we compared the generalizable models with models that were developed using the same 84 potential predictors, plus 49 additional infiltration questionnaire predictors that were specific to the 2-week I/O sampling period (henceforth “2-week specific models”). This comparison provided information on the loss of predictive power introduced by a lack of temporal specificity in the generalizable models. Second, after the predictive models were developed, we added possible indoor air pollution sources (presence of pilot lights, use of candles or incense, use of a humidifier, or participant report that the home had been smoky from cooking) to the models to determine if these sources were associated with higher I/O sulfur ratios. Finally, we compared I/O sulfur ratios with the corresponding PM_2.5_ ratios. A sulfur ratio that exceeds the corresponding PM_2.5_ ratio indicates *a*) measurement imprecision, *b*) an indoor sulfur source, or *c*) overestimation of PM_2.5_ *F*_inf_, possibly due to the loss of volatile PM_2.5_ species as the PM moves indoors (Sarnat et al. 2006).

## Results

The 353 homes that underwent I/O sampling were generally representative of the MESA Air cohort [see Supplemental Material, Table 1 (http://dx.doi.org/10.1289/ehp.1104447)]. Of the 353 homes, 173 were monitored twice, with most of those (119, 69%) monitored in different seasons. Because homes where a smoker was reported to reside were excluded from home sampling, indoor smoking in the past year (by residents or visitors) was reported more commonly among the full cohort (17%) than among the home sampling subgroup (4%). The home sampling subgroup was also overrepresented by single-family/free-standing homes (68%) relative to the full cohort (55%). Use of central AC was very similar between the two groups but varied widely among communities, ranging from 6% of MESA Air homes in New York City to 88% in Winston-Salem. Window opening patterns and air cleaner use were also very similar between the full cohort and the home sampling subgroup.

**Table 1:** Predictors in community- and season-specific Finf models.

Finf IQR	Predictor variable									Model R2
	Community	n	Building	Climate	Heat/AC	Window	Air cleaner	Interaction		
Warm season (> 18°C)															
Baltimore	39		0.39		—		> 25°C and home has central AC (–)		Central AC used almost daily in July (–)		Any windows open in July (+)	—	—		0.67
Chicago	28		0.35		—		—		Central AC used more than half time in July (–)		Any windows open in July (+)	—	—		0.78
Los Angeles	53		0.18		Free standing (+)		—		Central AC usea (–)		All windows open in July (+)	—	—		0.62
New York	26		0.10		Incomeb,c (–)		—		—		Windows open > half time in July (+)	—	—		0.51
Rockland County	11		0.39		—		—		Central AC used more than half time in July (–)		—	—	—		0.46
St. Paul	23		0.49		—		Temperatureb,d (+)		Home has central ACd (–)		Any windows open in July (+)	—	Temperature × central AC (–)		0.72
Winston-Salem	39		0.23		—		> 25°C and home has central AC (–)		Central AC used almost daily in July (–)		Windows open > half time in July (+)	—	—		0.51
Cold season (≤ 18°C)															
Baltimore	48		0.21		—		Temperatureb (+)		< 0°C and home has forced-air heat (–)		—	Air cleaner/filter used in the home (–)	—		0.54
Chicago	40		0.15		Free standing (–)		Temperatureb (+)		—		Any windows open in January (+)	HEPA or ESP used more than half time (–)	—		0.35
Los Angeles	80		0.20		—		Temperatureb (+)		Home has central AC (–)		Windows open > half time in July (+)	—	—		0.44
New York	24		0.26		—		Temperatureb (+)		—		—	—	—		0.23
Rockland County	12		0.20		—		—		—		—	—	—		—
St. Paul	56		0.27		—		Temperatureb (+)		Home has forced-air heat (–)		All windows open in January (+)	—	—		0.49
Winston-Salem	47		0.22		Home has a garage (–)		Temperatureb (+)		—		—	HEPA filter used almost daily (–)	—		0.33
Abbreviations: —, No statistically significant predictor; ESP, electrostatic precipitator; HEPA, high-efficiency particulate air filter; IQR, interquartile range. The direction of the coefficient is shown in parentheses. All variables are binary unless otherwise noted. aOrdinal variable. bContinuous variable. cIncome is assumed to be a surrogate for quality of construction, building materials, and so forth. dIncluded as main effects of interaction term.															

Two-week average *F*_inf_ varied between communities and between seasons within communities (Figure 1). The mean ± SD *F*_inf_ across all communities and seasons was 0.62 ± 0.21. Community-specific means ranged from 0.47 ± 0.15 in Winston-Salem to 0.82 ± 0.14 in New York. With the exception of Winston-Salem, where approximately 90% of homes used central AC in summer and window opening was infrequent, mean values of *F*_inf_ were generally greater during the warm season. Across all observations, PM_2.5_ of outdoor origin contributed roughly 80% of the indoor PM_2.5_ concentration in these homes. Summary statistics for PM_2.5_ and sulfur concentrations are presented in the Supplemental Material, Table 2 (http://dx.doi.org/10.1289/ehp.1104447).

**Table 2:** Season-specific Finf models combining data from all communities.

Questionnaire source for predictora	CV						
	R2			Season			Overall		
	Predictor	β (SE)	p-Value	Partial	Model	R2	RMSE	R2	RMSE
Generalizable modelb																0.60		0.13
Warm season (n = 219)										0.70		0.68		0.14				
Intercept		NA		0.72 (0.03)		< 0.01		NA										
Central AC used > half time in past July		Main		–0.22 (0.03)		< 0.01		0.560										
Windows open ≥ half time in past summer		Main		0.15 (0.02)		< 0.01		0.080										
Central AC used > half time in past July and 2-week average outdoor temperature > 23°C		Main		–0.16 (0.04)		< 0.01		0.051										
Central AC used a few days in past July		Main		–0.10 (0.03)		< 0.01		0.013										
2-week average outdoor temperature > 23°Cc		NA		0.01 (0.03)		0.75		0.000										
Cold season (n = 307)										0.49		0.47		0.13				
Intercept		NA		0.52 (0.02)		< 0.01		NA										
2-week average outdoor temperature (°C)		NA		0.01 (0.00)		< 0.01		0.222										
Home has forced-air heat		Main		–0.12 (0.02)		< 0.01		0.166										
Windows open ≥ half time in past summer		Main		0.08 (0.02)		< 0.01		0.069										
Home has double pane windows		Main		–0.05 (0.02)		< 0.01		0.023										
Windows open ≥ half time in past winter		Main		0.05 (0.02)		< 0.01		0.014										
2-week specific modeld																0.66		0.13
Warm season (n = 198)										0.75		0.74		0.12				
Intercept		NA		0.63 (0.03)		< 0.01		NA										
Central AC used at all in past July		Main		–0.16 (0.02)		< 0.01		0.563										
Central AC used ≥ 6 days during sampling		Infiltration		–0.11 (0.03)		< 0.01		0.102										
Windows open ≥ 11 days during sampling		Infiltration		0.16 (0.03)		< 0.01		0.048										
Windows open ≥ half time in past summer		Main		0.09 (0.02)		< 0.01		0.025										
Windows open 6–10 days during sampling		Infiltration		0.10 (0.03)		< 0.01		0.014										
Cold season (n = 269)										0.56		0.53		0.13				
Intercept		NA		0.54 (0.02)		< 0.01		NA										
Windows open ≥ 11 days during sampling		Infiltration		0.09 (0.02)		< 0.01		0.242										
2-week average outdoor temperature (°C)		NA		0.01 (0.00)		< 0.01		0.131										
Home has forced-air heat		Main		–0.11 (0.02)		< 0.01		0.119										
Central AC used ≥ 11 days during sampling		Infiltration		–0.17 (0.05)		< 0.01		0.025										
Home has double pane windows		Main		–0.04 (0.02)		< 0.01		0.019										
Windows open ≥ half time in past summer		Main		0.05 (0.02)		< 0.01		0.014										
HEPA or ESP used ≥ 11 days during sampling		Infiltration		–0.11 (0.04)		< 0.01		0.013										
NA, not applicable. aThe specific questions used to derive the predictors are listed in the Supplemental Material [Tables 3, 4 (http://dx.doi.org/10.1289/ehp.1104447)]. bIncludes only variables available for the full cohort. cIncluded as a main effect in a significant interaction term. dIncludes both variables available for the full cohort and variables specific to the 2-week sampling period.																		

In the preliminary analysis of community- and season-specific predictors, we found that the most consistent *F*_inf_ predictors during the warm season were variables related to window opening frequency and use of central AC (Table 1). Outdoor temperature was the most consistent *F*_inf_ predictor during the cold season.

Window opening and use of central AC were also important in our generalizable warm-season model. The warm-season model included three variables related to central AC use and one variable related to window opening frequency and had a CV *R*^2^ of 0.68 and a CV RMSE of 0.14 (Table 2). The single most important warm-season predictor was the use of central AC more than half of the time in the past July (partial *R*^2^ = 0.56). The cold-season generalizable model included as predictors outdoor temperature, the presence of forced-air heat, the presence of double pane windows, and two window opening frequency variables and had CV *R*^2^ and RMSE of 0.47 and 0.13, respectively. Outdoor temperature (partial *R*^2^ = 0.22) and the presence of forced-air heat (partial *R*^2^ = 0.17) were the most important cold-season predictors. Variance inflation factors for the predictors in the generalizable warm- and cold-season models were < 3.3 and < 1.2, respectively. In addition to two predictors based on outdoor temperature, a total of seven unique questionnaire-based predictors were used in the generalizable models (Table 2). These seven predictors were derived from a total of nine unique questions, which are provided in the Supplemental Material [Table 3 (http://dx.doi.org/10.1289/ehp.1104447)].

**Figure 2:**
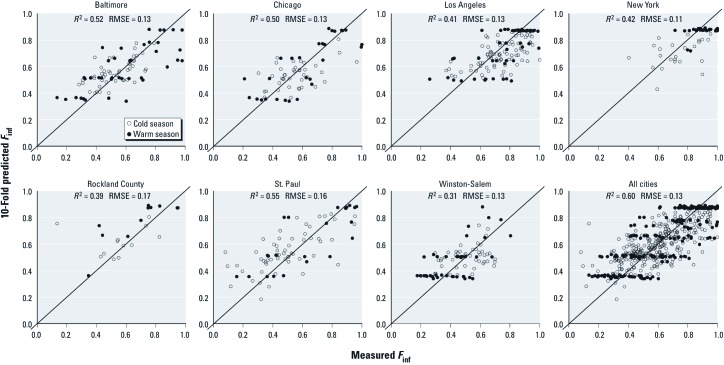
Comparisons of measured *F*_inf_ (*x*-axes) with values predicted from a 10-fold CV (*y*-axes) for the generalizable models shown in Table 2. White and black circles represent cold and warm seasons, respectively; lines represent 1:1.

Across seasons, the overall generalizable model CV *R*^2^ and RMSE were 0.60 and 0.13, respectively (Table 2, Figure 2). The generalizable models performed similarly when evaluated on individual communities (Figure 2). The predictions were less variable than the measurements; the models consistently overestimated *F*_inf_ < 0.3 and underestimated *F*_inf_ > 0.9 (Figure 2). Results were very similar when using a more conservative leave-one-community-out CV model assessment approach [see Supplemental Material, Figure 2 (http://dx.doi.org/10.1289/ehp.1104447)].

The generalizable models performed nearly as well as the 2-week specific models (overall CV *R*^2^ = 0.66; CV RMSE = 0.13; Table 2), suggesting that the lack of temporal specificity in the main questionnaire did not substantially reduce model performance (there were fewer observations for the 2-week specific models because some participants did not complete the infiltration questionnaire; the generalizable model results were similar when applied to participants for which infiltration questionnaire data were available: overall CV *R*^2^ = 0.62; CV RMSE = 0.13). Presence of pilot lights, use of candles or incense, self-reported smoky periods from cooking, and use of a humidifier were reported during 72%, 18%, 14%, and 4% of the I/O sampling observations, respectively. None of these were significant predictors of *F*_inf_ (data not shown).

The agreement between responses on the main questionnaire and responses on the time-specific infiltration questionnaire varied across behaviors. For window opening there was reasonable agreement (Kendall’s tau-b ≥ 0.46), whereas the agreement for central AC use in summer was very good (Kendall’s tau-b = 0.70). For HEPA filters or electrostatic precipitator use, which was reported relatively infrequently, the agreement was poorer (Kendall’s tau-b = 0.21).

**Figure 3:**
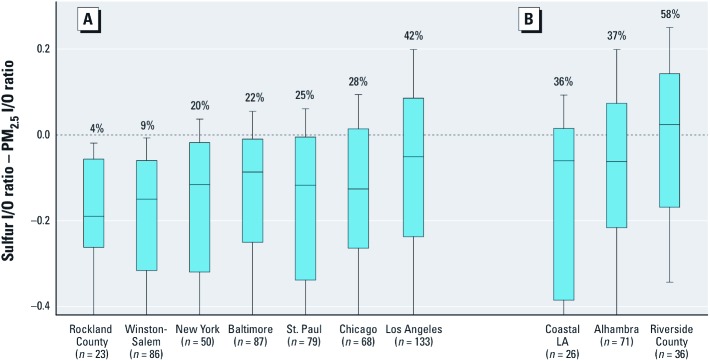
Distributions of differences between sulfur I/O ratio and PM_2.5_ I/O ratio in all seven study communities (*A*) and the Los Angeles study community stratified by recruitment area (*B*). The percentages of observations with differences > 0 are shown for each community. Lines in boxes represent median values; boxes represent 25th and 75th percentiles; whiskers represent 10th and 90th percentiles; outliers are not shown.

The frequency with which sulfur I/O ratios exceeded corresponding PM_2.5_ ratios ranged from 4% in Rockland County to 42% in Los Angeles (Figure 3A). Differences were observed across the Los Angeles study region after stratifying homes into those recruited from the main MESA study (Alhambra) and the two MESA Air new recruitment areas (coastal Los Angeles and Riverside County). In coastal Los Angeles, Alhambra, and Riverside County, 36%, 37%, and 58% of the observations, respectively, had a sulfur I/O ratio that exceeded the corresponding PM_2.5_ ratio (Figure 3B).

## Discussion

This is the first data set developed specifically to predict *F*_inf_ for exposure assessment in a large cohort study. We found considerable variation in *F*_inf_, suggesting that differences in *F*_inf_ may be an important source of heterogeneity in exposure to PM_2.5_ of outdoor origin, even in studies focused on within-city gradients. The models explained a substantial portion of this variation using relatively easily collected and intuitive predictors.

Our generalizable models explained 60% of the variance in 2-week averaged *F*_inf_ (RMSE = 0.13). To our knowledge, the only other attempt to model *F*_inf_ in a large number of homes in multiple communities was the Relationships of Indoor, Outdoor, and Personal Air (RIOPA) study, which modeled 114 *F*_inf_ measurements from Houston, Texas; Elizabeth, New Jersey; and Los Angeles (Meng et al. 2009). Despite including measured air exchange rate in their model, the model-based *R*^2^ was 0.49. The partial *R*^2^ of air exchange was 0.36, whereas central AC and outdoor temperature made small contributions to the *R*^2^. Other attempts to model *F*_inf_ have relied on relatively small data sets and have had mixed success (Clark et al. 2010; Hystad et al. 2009; Koenig et al. 2005).

Most air pollution epidemiologic studies use outdoor concentration as a surrogate for exposure to pollution of outdoor origin; only a few panel studies have explicitly considered *F*_inf_ as part of the exposure assessment (Allen et al. 2008; Ebelt et al. 2005; Koenig et al. 2005). Outdoor-source exposure is a function of outdoor concentration and an attenuation factor. The magnitude of attenuation is a weighted average of *F*_inf_ and time spent outdoors (Sheppard et al. 2011), and because people spend most of their time indoors (Klepeis et al. 2001), *F*_inf_ is the most important component of outdoor attenuation. When outdoor concentration is used as a surrogate for exposure the health effect parameter estimated is the product of the toxicity and outdoor attenuation (Sheppard et al. 2011; Zeger and Diggle 2001). Using outdoor-source exposure in place of outdoor concentration in epidemiologic studies should result in less attenuated health effect parameter estimates (Koenig et al. 2005). Although a reduction in classical-like measurement error (Szpiro et al. 2011) could contribute, the dominant reason for the decreased attenuation is the change in the target parameter. This understanding is consistent with results from time-series studies suggesting that AC (as a surrogate for *F*_inf_) may be an important modifier of the relationship between outdoor concentrations and health. For example, Bell et al. (2009) reported that communities with more prevalent AC use had lower PM effects on cardiovascular hospitalizations, and that central AC prevalence explained 17% of the between-community variability in PM_2.5_ effect estimates. However, central AC is only one of several factors influencing *F*_inf_, and interpretation of effect modification by (ecologic) AC prevalence is problematic (Vedal 2009).

Our model predictors are consistent with previous findings. Studies have found lower *F*_inf_ in homes with central AC (Clark et al. 2010; Meng et al. 2009), which may influence *F*_inf_ by discouraging window opening and/or by increasing PM deposition on filters or in air ducts (Howard-Reed et al. 2003). Window opening increases *F*_inf_ by increasing the home’s air exchange rate. For example, Wallace et al. (2002) found that air exchange rates in a Reston, Virginia, house averaged 0.65/hr over a 1-year period but increased to 2/hr with windows open. The most important predictor of cold-season *F*_inf_ was outdoor temperature, consistent with our results from Seattle, Washington (Koenig et al. 2005). This variable probably contributed additional information on window opening beyond that captured by questionnaire. Forced-air heat was associated with lower *F*_inf_, presumably also due to deposition of PM on filters or in air ducts (Howard-Reed et al. 2003).

In exploratory community-specific *F*_inf_ models, we found consistency in predictors among communities (Table 1). This result, and the small sample sizes in individual communities, motivated us to develop models across all communities. This approach potentially allows our models to be used outside of the MESA Air cohort. The similarity between the 10-fold (Figure 2) and leave-one-community-out CV results [Supplemental Material, Figure 2 (http://dx.doi.org/10.1289/ehp.1104447)] suggests that the models predict important sources of variability across communities. Moreover, the diverse communities in MESA Air suggest the potential for applying this *F*_inf_ model to other communities in future epidemiologic studies, although the generalizability of our models to other communities will need to be determined using independent observations.

The generalizability of our models is enhanced by the types of questions that were used to derive many of the predictors (for questions, see Supplemental Material, Table 3 (http://dx.doi.org/10.1289/ehp.1104447). In chronic exposure studies it is not feasible to obtain temporally resolved information on participant behaviors over the entire duration of follow-up. Therefore, we asked MESA Air participants questions about typical behavior during summer and winter and found that responses to these questions agreed reasonably well with actual behaviors during the 2-week I/O sampling periods. This was particularly true for central AC use, which was the most important warm-season predictor of *F*_inf_. Because of this agreement, our generalizable models performed nearly as well as models using predictors specific to the 2-week sampling period (Table 2).

*F*_inf_ varies with PM size, with a maximum for PM of approximately 0.1–0.5 μm (Sarnat et al. 2006). Sarnat et al. (2002) found that sulfur PM, which is in the 0.2–0.7 μm size range, adequately traced PM_2.5_ infiltration but cautioned that sulfur overestimates *F*_inf_ for PM < 0.06 μm or > 0.7 μm. *F*_inf_ also varies with PM composition. Because it is nonvolatile, sulfur may overestimate PM_2.5_ *F*_inf_ when the outdoor PM_2.5_ contains large quantities of volatile species. Sarnat et al. (2006) compared *F*_inf_ for PM_2.5_, black carbon (a nonvolatile component), and nitrate (a volatile component) in Los Angeles homes. The median *F*_inf_ for PM_2.5_ (0.48) fell between those for nitrate (0.18) and black carbon (0.84), indicating a loss of nitrate indoors. The indoor volatilization of nitrate may explain the sulfur I/O ratios that exceeded PM_2.5_ ratios in several of our Los Angeles homes and the spatial pattern of those exceedances. Nitrate contributions to PM_2.5_ in greater Los Angeles [31% at downtown Los Angeles and 46% at Rubidoux (Kim and Hopke 2007)] are greater than in other MESA Air communities [23% in Baltimore (Ogulei et al. 2005), 20% in Chicago (Rizzo and Scheff 2007), 8–18% in New York (Qin et al. 2006), and 6–9% in Winston-Salem (Aneja et al. 2006)]. Nevertheless, although it may overestimate PM_2.5_ in some settings, sulfur is currently the best tracer of PM_2.5_ *F*_inf_. The incorporation of *F*_inf_ into the exposure assessment in MESA Air represents a significant advance over previous studies that have not considered *F*_inf_ and thus assumed an unrealistic constant relationship between outdoor concentrations and personal exposures.

## Conclusions

Our finding of wide variation in residential PM_2.5_ *F*_inf_ suggests that it is an important source of exposure heterogeneity in epidemiologic studies of exposure to PM_2.5_ of outdoor origin. Using a large, unique data set collected specifically to predict infiltration in an ongoing cohort study, we developed intuitive models that explain a substantial portion of infiltration variation using relatively easily obtained predictors. Using these models, MESA Air will be the first large epidemiologic study to incorporate variation in residential *F*_inf_ into an exposure assessment. This will provide more variable estimates of exposure and potentially allow for more accurate and precise estimates of the cardiovascular risks of outdoor-generated PM_2.5_.

## Supplemental Material

(152 KB) PDF
