# Supplementary material for: Modeling the Residential Infiltration of Outdoor PM2.5 in the Multi-Ethnic Study of Atherosclerosis and Air Pollution (MESA Air)
Source: Environ Health Perspect. 2012 Feb 22;120(6):824–30. doi: 10.1289/ehp.1104447 (PMC3385439; doi:10.1289/ehp.1104447)
Supplement: (152 KB) PDF [file ehp.1104447.s001.pdf]

Supplemental Material:

Modeling the Residential Infiltration of Outdoor $PM_{2.5}$ in the Multi-Ethnic Study of Atherosclerosis and Air Pollution (MESA Air)

Ryan W. Allen[1*], Sara D. Adar[2], Ed Avol[3], Martin Cohen[4], Cynthia L. Curl[4], Timothy Larson[4,5], L.-J. Sally Liu[4,6], Lianne Sheppard[4,7], and Joel D. Kaufman[4,8,9]

[1]Faculty of Health Sciences, Simon Fraser University, Burnaby, BC, Canada
[2]Department of Epidemiology, University of Michigan, Ann Arbor, MI, USA
[3]Department of Preventive Medicine, University of Southern California, Los Angeles, CA, USA
[4]Department of Environmental & Occupational Health Sciences, University of Washington, Seattle, WA, USA
[5]Department of Civil and Environmental Engineering, University of Washington, Seattle, WA, USA
[6]Institute of Social and Preventive Medicine, University of Basel, Basel, Switzerland
[7]Department of Biostatistics, University of Washington, Seattle, WA, USA
[8]Department of Epidemiology, University of Washington, Seattle, WA, USA
[9]Deparment of Medicine, University of Washington, Seattle, WA, USA

Table of Contents for Supplementary Material

	Page
Methods	
Data Reduction	3
Calculation of Contributions to Indoor Concentrations	3
Model Development	4
References	5
Table 1: Comparison of selected home and resident characteristics between the full MESA Air cohort and the subgroup selected for home indoor/outdoor (I/O) sampling.	6
Table 2: Mean (± SD) 2-week $PM_{2.5}$ concentrations and estimated indoor and outdoor contributions to indoor $PM_{2.5}$ concentrations by community and season.	7
Table 3: Main MESA Air Questionnaire questions used to derive predictors in the generalizable and 2-week specific infiltration efficiency models.	8
Table 4: Infiltration Questionnaire questions used to derive predictors in the 2-week specific infiltration efficiency models.	9
Figure 1. Infiltration efficiency vs. average outdoor temperature during the 2-week sampling period among a) homes not using air conditioning and b) homes using air conditioning.	10
Figure 2: Comparisons of measured infiltration efficiencies (x-axes) with values predicted from a leave-one-community out cross validation (y-axes) for the generalizable models shown in Table 2.	11

Methods

Data Reduction

After QA/QC checks for pump failures, dropped/torn filters, etc., there were 573 valid I/O sulfur pairs. To minimize the influence of indoor sulfur sources, we excluded 22 observations where the participant reported that smoking had occurred in the home during the 2-week period, and 6 cold season observations from homes with kerosene heaters (Koutrakis et al. 1992). We also removed 11 observations where the I/O sulfur ratio was greater than 1.05, which indicated an indoor sulfur source. We included ratios between 1.00 and 1.05 to account for imprecision in the sulfur measurements. We also excluded 6 observations where the participants reported that the home was "smoky from cooking" for ≥ 10 hours during the 2-week sampling period, since window opening and other behaviors during such periods may not represent typical conditions. To minimize the influence of extreme values on the F_{inf} models, we also removed one distant outlier (F_{inf} more than 3 interquartile ranges below the community- and season-specific 25th percentile) in Los Angeles and New York. These exclusions left 526 I/O sulfur pairs (from 353 homes) for analysis.

Calculation of Contributions to Indoor Concentrations

For each valid F_{inf} observation, we estimated the infiltrated ($PM_{2.5}^{\text{inf}}$) and indoor-generated ($PM_{2.5}^{ig}$) contributions to the 2-week average indoor $PM_{2.5}$ concentrations based on the measured $PM_{2.5}$ concentrations outdoors ($PM_{2.5}^{outdoor}$) and indoors ($PM_{2.5}^{indoor}$) and the home-specific estimate of F_{inf} (Allen et al. 2004):

$$PM_{2.5}^{\text{inf}} = F_{\text{inf}} \text{ x } PM_{2.5}^{outdoor} \quad (1)$$

and

$$PM_{2.5}^{ig} = PM_{2.5}^{indoor} - PM_{2.5}^{\text{inf}} \quad (2)$$

When $PM_{2.5}^{\inf} > PM_{2.5}^{indoor}$, we set $PM_{2.5}^{\inf} = PM_{2.5}^{indoor}$ and $PM_{2.5}^{ig} = 0$.

Model Development

We developed models using two approaches. In the first approach we 1) calculated the correlations between each predictor and $F_{\inf}$, 2) offered the significantly ($p<0.1$) correlated predictors into a stepwise linear regression ($p<0.30$ to enter; $p<0.10$ to remain) with $F_{\inf}$ as the dependent variable, and 3) removed predictors that contributed less than 0.01 to the model R^2. In the second approach, we 1) calculated the correlations between each predictor and $F_{\inf}$, 2) entered the highest-correlated predictor into a model with $F_{\inf}$ as the dependent variable, 3) calculated the model residuals, 4) calculated the correlations between the model residuals and all remaining predictors, 5) added the highest-correlated predictor as an additional predictor in the model with $F_{\inf}$ as the dependent variable, and 6) repeated steps 3-5 until the model included all variables with $p<0.10$ that contributed at least 0.01 to the model R^2. Under both methods, we only included predictors for which the coefficient's sign was consistent with physical processes (e.g., positive coefficients for window opening). Since the models were developed to predict $F_{\inf}$, we did not account for possible dependence between measurements made in the same home or in the same community. In the preliminary community-specific models we required that every variable have at least 4 non-zero observations to be included in the model.

References

Allen R, Wallace L, Larson T, Sheppard L, Liu LJS. 2004. Estimated hourly personal exposures to ambient and nonambient particulate matter among sensitive populations in Seattle, Washington. J Air Waste Manag Assoc 54(9):1197-1211.

Koutrakis P, Briggs SLK, Leaderer BP. 1992. Source Apportionment Of Indoor Aerosols In Suffolk And Onondaga Counties, New-York. Environ Sci Technol 26(3):521-527.

Supplemental Material Table 1. Comparison of selected home and resident characteristics between the full MESA Air cohort and the subgroup selected for home indoor/outdoor (I/O) sampling.

Community	Total number of homes		Single family / free standing home[a]		Central AC used in the past July[b]		Usually had windows open in the past summer[c]		HEPA filter / electrostatic precipitator used[d]		Smoking inside the home in the past year[e]	
	All Homes	I/O Homes	% of All Homes	% of I/O Homes	% of All Homes	% of I/O Homes	% of All Homes	% of I/O Homes	% of All Homes	% of I/O Homes	% of All Homes	% of I/O Homes
Baltimore	721	56	62	64	64	54	64	68	6	7	19	5
Chicago	1,146	46	41	54	56	67	75	74	16	24	16	0
Los Angeles	1,176	89	71	78	42	45	89	84	6	8	10	4
New York	1,103	39	7	3	6	0	90	92	3	5	20	8
Rockland	100	18	71	72	55	39	89	94	11	17	13	6
St. Paul	879	50	71	82	47	54	86	80	8	6	21	4
Winston-Salem	890	55	89	98	88	93	36	27	12	16	21	0
Total	6,015	353	55	68	49	56	75	72	9	11	17	4

Based on responses provided on the MESA Air Questionnaire at study entry. Questions were worded as follows:
[a] "What type of building do you live in?" = Single-family or free-standing.
[b] "What type of air conditioning does your residence have?" = Central AC + "How often was the air conditioning used in the past July?" ≥ A few days a month
[c] "How many windows did you usually have open in the past summer?" = All or Some
[d] "What type of air cleaner/filter is used in your residence?" = HEPA filter and/or electrostatic precipitator
[e] "Did anyone smoke in your residence in the past 12 months (this includes you)?" = Yes

Supplemental Material Table 2. Mean (± SD) 2-week $PM_{2.5}$ concentrations and estimated indoor and outdoor contributions to indoor $PM_{2.5}$ concentrations by community and season.

Community	Season[a]	Observations (Homes)[b]	Outdoor Sulfur (μg/m^3)	Indoor Sulfur (μg/m^3)	Outdoor $PM_{2.5}$ (μg/m^3)	Indoor $PM_{2.5}$ (μg/m^3)	Indoor-Generated Indoor $PM_{2.5}$ (μg/m^3)	Infiltrated Indoor $PM_{2.5}$ (μg/m^3)	Infiltrated $PM_{2.5}$ Contribution to Indoor $PM_{2.5}$ (%)
Baltimore	Cold	48 (41)	1.19 ± 0.24	0.63 ± 0.23	11.8 ± 2.5	7.9 ± 2.9	1.9 ± 2.3	6.0 ± 2.0	80.3 ± 20.1
	Warm	39 (36)	2.22 ± 0.52	1.29 ± 0.56	16.8 ± 3.7	12.5 ± 5.7	2.7 ± 3.6	9.7 ± 4.3	80.3 ± 17.9
Chicago	Cold	40 (33)	1.00 ± 0.25	0.56 ± 0.24	13.3 ± 3.3	9.1 ± 4.2	2.4 ± 4.3	6.7 ± 2.0	80.9 ± 22.8
	Warm	28 (27)	1.39 ± 0.37	0.87 ± 0.45	14.0 ± 3.0	12.1 ± 6.0	3.6 ± 4.6	8.5 ± 3.7	74.8 ± 19.5
Los Angeles	Cold	80 (71)	0.87 ± 0.59	0.66 ± 0.51	17.2 ± 8.1	13.9 ± 8.6	2.8 ± 4.9	11.1 ± 5.7	84.9 ± 18.7
	Warm	53 (52)	1.51 ± 0.53	1.19 ± 0.55	16.0 ± 2.6	13.7 ± 3.7	1.5 ± 2.1	12.2 ± 3.5	89.4 ± 12.5
New York	Cold	24 (23)	1.29 ± 0.56	0.91 ± 0.24	16.2 ± 6.8	16.4 ± 10.7	5.4 ± 9.4	11.0 ± 2.8	76.1 ± 22.5
	Warm	26 (23)	1.68 ± 0.47	1.52 ± 0.46	15.7 ± 3.2	17.3 ± 6.5	3.2 ± 6.6	14.2 ± 3.5	86.5 ± 17.8
Rockland	Cold	12 (11)	0.89 ± 0.12	0.49 ± 0.15	9.8 ± 2.8	7.7 ± 2.7	2.3 ± 2.0	5.4 ± 1.9	71.3 ± 19.6
	Warm	11 (11)	2.05 ± 0.47	1.41 ± 0.56	17.0 ± 3.8	14.3 ± 6.5	3.2 ± 4.7	11.1 ± 4.1	79.4 ± 17.9
St. Paul	Cold	56 (45)	0.69 ± 0.15	0.35 ± 0.18	10.0 ± 3.5	7.2 ± 5.9	2.8 ± 5.1	4.4 ± 2.0	72.5 ± 23.6
	Warm	23 (23)	0.90 ± 0.32	0.56 ± 0.30	9.8 ± 1.7	7.3 ± 2.6	1.2 ± 1.3	6.1 ± 3.1	82.4 ± 19.6
Winston-Salem	Cold	47 (40)	1.18 ± 0.27	0.60 ± 0.22	12.6 ± 2.8	9.3 ± 4.1	3.0 ± 3.4	6.3 ± 2.4	72.4 ± 20.7
	Warm	39 (36)	2.38 ± 0.63	1.00 ± 0.43	18.6 ± 3.8	11.9 ± 5.0	4.1 ± 4.1	7.8 ± 3.1	69.6 ± 21.1
All	Cold	307 (264)	0.98 ± 0.43	0.60 ± 0.35	13.5 ± 5.8	10.4 ± 7.0	2.8 ± 4.8	7.6 ± 4.3	78.3 ± 21.5
	Warm	219 (208)	1.76 ± 0.69	1.12 ± 0.55	15.8 ± 3.9	12.8 ± 5.6	2.7 ± 4.0	10.1 ± 4.3	80.7 ± 18.9

[a] Cold and warm seasons defined as ≤18 ^{o}C and >18 ^{o}C, respectively, during the 2-week I/O sampling period
[b] Some homes were monitored twice in the same season.

Supplemental Material Table 3. Main MESA Air Questionnaire questions used to derive predictors in the generalizable and 2-week specific infiltration efficiency models.

Predictor	Questions	Response(s)[a]
**Central AC used a few days in the past July**	Do you use air conditioning in your residence?	Yes
	What type of air conditioning does your residence have?	Central A/C
	How often was the air conditioning used in the past July?	A few days a month
**Central AC used > ½ time in the past July**	Do you use air conditioning in your residence?	Yes
	What type of air conditioning does your residence have?	Central A/C
	How often was the air conditioning used in the past July?	More than half the days of the month, but less than daily OR Almost daily (thermostat use also)
**Central AC used at all in the past July**	Do you use air conditioning in your residence?	Yes
	What type of air conditioning does your residence have?	Central A/C
	How often was the air conditioning used in the past July?	A few days a month OR More than half the days of the month, but less than daily OR Almost daily (thermostat use also)
**Home has forced air heat**	What are the heating sources used in your residence? Please check all that are used at least once a month.	Forced air (vents)
**Home has double pane windows**	Does your residence have double pane windows?	Yes
**Windows open ≥ ½ time in the past summer**	In summer (Jun – Aug) how many windows did you usually have open?	All OR Some
	In summer (Jun – Aug) how often did you open windows?	More than half the days of the month, but less than daily OR Almost daily
**Windows open ≥ ½ time in the past winter**	In winter (Dec – Feb) how many windows did you usually have open?	All OR Some
	In winter (Dec – Feb) how often did you open windows?	More than half the days of the month, but less than daily OR Almost daily

[a]Homes with these responses were coded as 1; homes with other responses were all coded as 0.

Supplemental Material Table 4. Infiltration Questionnaire questions used to derive predictors in the 2-week specific infiltration efficiency models.

Predictor	Questions	Response(s)[a]
**Central AC used ≥ 6 days during sampling**	Does your home have air conditioning?	Yes
	What type of air conditioning does your residence have?	Central A/C
	How often did you use air conditioning in the past 12-14 days?	6 – 10 days OR 11-14 days
**Central AC used ≥ 11 days during sampling**	Does your home have air conditioning?	Yes
	What type of air conditioning does your residence have?	Central A/C
	How often did you use air conditioning in the past 12-14 days?	11-14 days
**HEPA or ESP used ≥ 11 days during sampling**	During the past 12-14 days, was an air cleaner/filter (stand-alone or central) used in your home?	Yes
	What kind of air cleaner did you use?	HEPA filter OR Electrostatic precipitator
	How often was the air cleaner/filter used in the past 12-14 days?	11-14 days
**Windows open 6-10 days during sampling**	During the past 12-14 days, how often did you have windows open?	6-10 days
**Windows open ≥ 11 days during sampling**	During the past 12-14 days, how often did you have windows open?	11-14 days

[a]Homes with these responses were coded as 1; homes with other responses were all coded as 0.

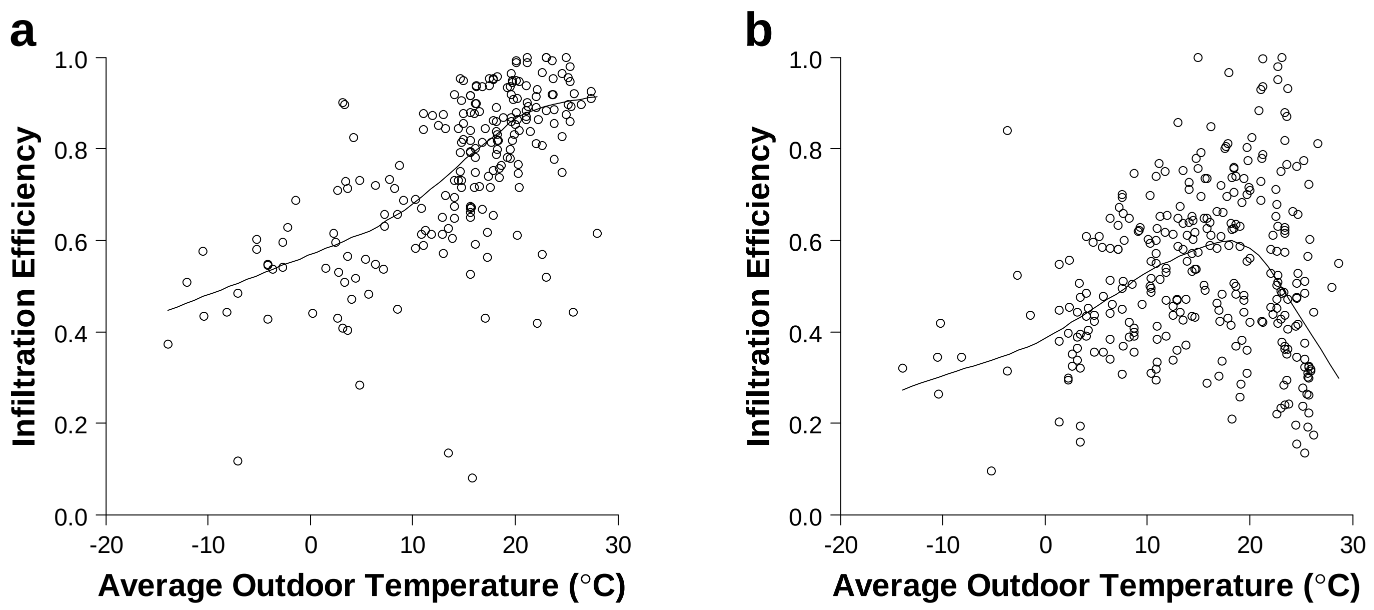

Supplemental Material Figure 1. Infiltration efficiency vs. average outdoor temperature during the 2-week sampling period among a) homes not using air conditioning and b) homes using air conditioning.

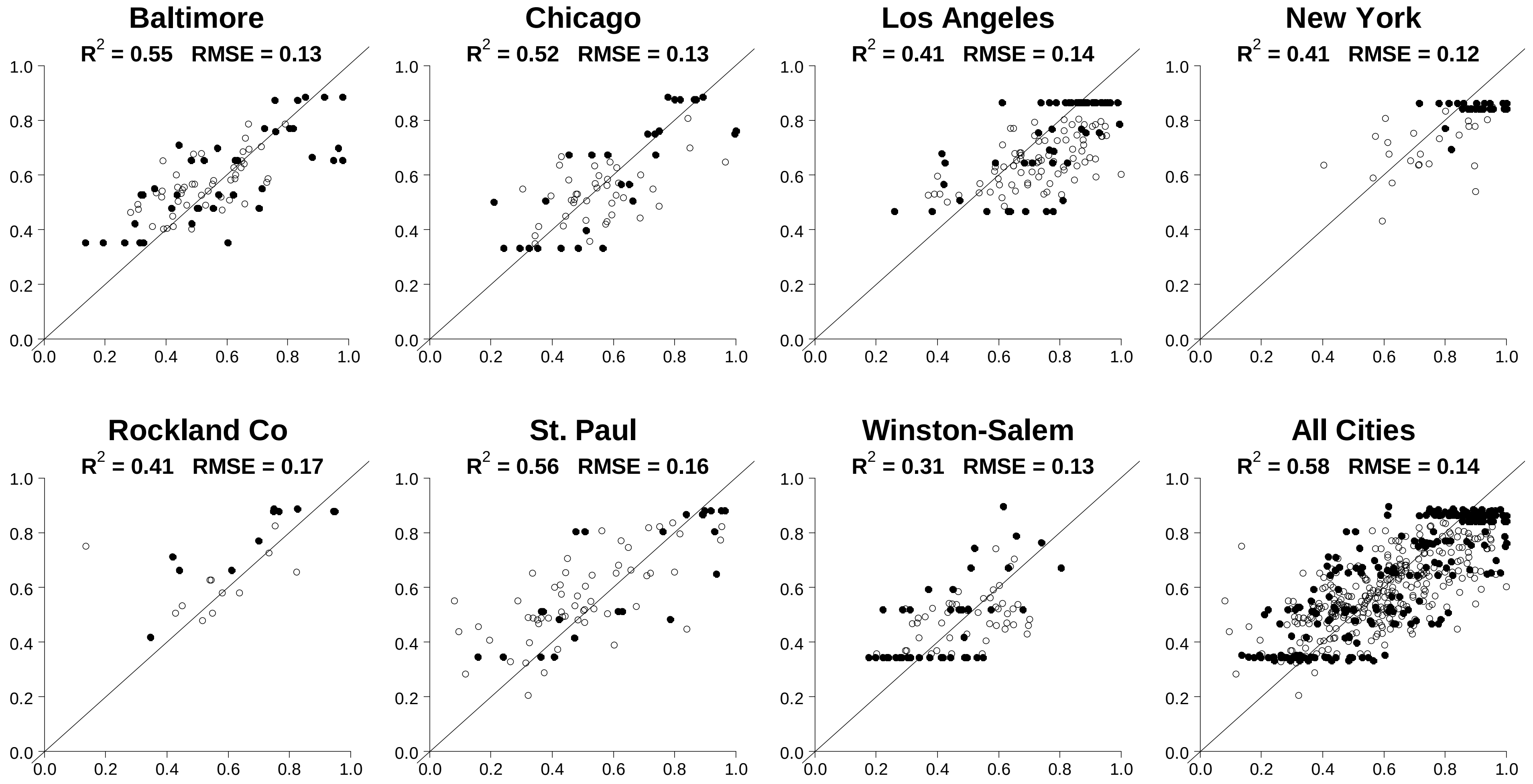

Supplemental Material Figure 2. Comparisons of measured infiltration efficiencies (x-axes) with values predicted from a leave-one-community out cross validation (y-axes) for the generalizable models shown in Table 2. White and black circles represent cold and warm seasons, respectively; lines represent 1:1.
